# Supplementary figures and images for: SOX7: Autism associated gene identified by analysis of multi-Omics data
Source: PLoS One. 2025 May 15;20(5):e0320096. doi: 10.1371/journal.pone.0320096 (PMC12080844; doi:10.1371/journal.pone.0320096)

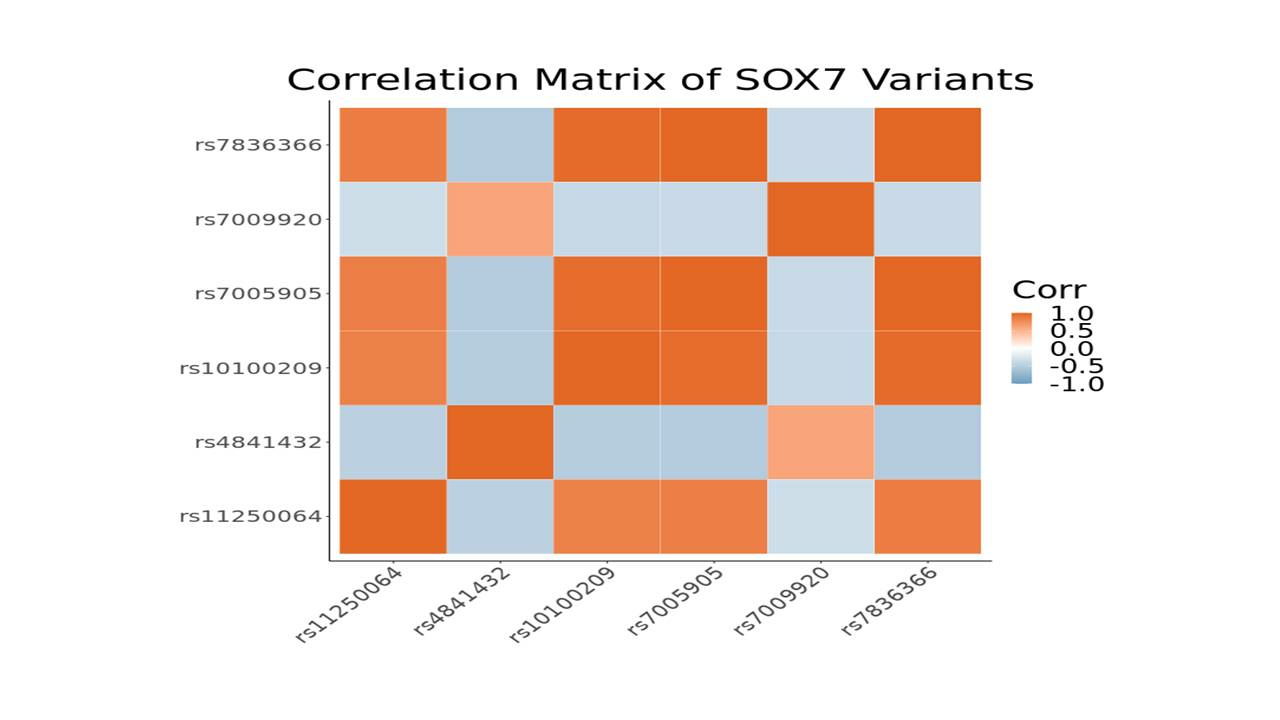

Supplement: S1 Fig — SNP rs7005905 and rs7836366, rs10100209 and rs7836366, and rs10100209 and rs7005905 have strong positive linkage disequilibrium (LD) (ρ > 0.5); rs4841432 has negative LD with other variants except for rs7009920. (TIF) [file pone.0320096.s001.tif]
