# Supplementary material for: SOX7: Autism associated gene identified by analysis of multi-Omics data
Source: PLoS One. 2025 May 15;20(5):e0320096. doi: 10.1371/journal.pone.0320096 (PMC12080844; doi:10.1371/journal.pone.0320096)
Supplement: S1 Table — (DOCX) [file pone.0320096.s002.docx]

**Supplementary Table 1.** Characteristics of participants and gene *SOX7* expression status in GSE211154 RNA-seq data.

| **Variable** | **Autism Cases (n=20)** | **Controls (n=19)** | **p-value*** |
| --- | --- | --- | --- |
| **Age at dealth (Year)**  **(Median [Inter-quartile range (IQR)])** | 17.5 (11.5) | 20 (11) | 0.44 |
| **Postmortem interval (IQR hour)** | 22.5 (5.8) | 15 (8.5) | 0.06 |
| **Sex (Male No. [%])** | 17 (85%) | 16 (84%) | 0.95 |
| **Race (No. [%])** |  |  |  |
| White | 16 (80%) | 12 (63.2%) | 0.25 |
| Black | 4 (20%) | 7 (36.8%) |  |
| **SOX7 in all samples (Median [IQR])** | 22.5 (14.25) | 16 (11) |  |
| Low expression (counts ≤median) (No. [%]) | 8 (40%) | 13 (68.4%) | 0.08 |
| High expression (count>median)(No. [%]) | 12 (60%) | 6 (31.6%) |  |
| **SOX7 in white samples (Median [IQR])** | 21 (18.5) | 14 (4.3) |  |
| Low expression (counts ≤median) (No. [%]) | 6 (37.5%) | 10 (83.3%) | 0.03 |
| High expression (count>median)(No. [%]) | 10 (62.5%) | 2 (16.7%) |  |
| Note: *p-value of Z test for each predictor is obtained from univariate logistic regression. | | | |
