# Supplementary material for: SOX7: Autism associated gene identified by analysis of multi-Omics data
Source: PLoS One. 2025 May 15;20(5):e0320096. doi: 10.1371/journal.pone.0320096 (PMC12080844; doi:10.1371/journal.pone.0320096)
Supplement: S2 Table — (DOCX) [file pone.0320096.s003.docx]

| **Supplementary Table 2.** 2-SMR results between ASD (Outcome) and *SOX7* expression (Exposure) | | | |
| --- | --- | --- | --- |
| **SOX7 Expressing Tissue** | **Beta** | **SE** | **P-value^1^** |
| Adipose Subcutaneous | 0.0387 | 0.0649 | 5.51e-01 |
| Adipose Visceral Omentum | 0.2084 | 0.1119 | 6.27e-02 |
| Adrenal Gland | 0.2808 | 0.0567 | **7.31e-07** |
| Artery Coronary | -0.3076 | 0.1021 | 2.59e-03 |
| Artery Tibial | 0.2372 | 0.1375 | 8.46e-02 |
| Brain Amygdala | 0.0275 | 0.0514 | 5.93e-01 |
| Brain Anterior cingulate cortex BA24 | -0.0183 | 0.0614 | 7.65e-01 |
| Brain Caudate basal ganglia | -0.0101 | 0.0708 | 8.86e-01 |
| Brain Cerebellar Hemisphere | 0.1054 | 0.0304 | **5.31e-04** |
| Brain Frontal Cortex BA9 | 0.0097 | 0.0381 | 7.98e-01 |
| Brain Hypothalamus | 0.1799 | 0.0514 | **4.71e-04** |
| Brain Putamen basal ganglia | -0.0225 | 0.0753 | 7.65e-01 |
| Brain Spinal cord cervical c-1 | 0.1997 | 0.0601 | **8.84e-04** |
| Brain Substantia nigra | -0.1037 | 0.0380 | 6.39e-03 |
| Cells Cultured fibroblasts | 0.1299 | 0.0582 | 2.58e-02 |
| Colon Sigmoid | 0.0453 | 0.0936 | 6.29e-01 |
| Esophagus Gastroesophageal Junction | 0.1054 | 0.0837 | 2.08e-01 |
| Esophagus Muscularis | 0.0192 | 0.1066 | 8.57e-01 |
| Lung | -0.5016 | 0.1290 | **1.01e-04** |
| Muscle Skeletal | 0.3941 | 0.1186 | **8.89e-04** |
| Nerve Tibial | -0.1195 | 0.0724 | 9.89e-02 |
| Ovary | -0.1701 | 0.0731 | 1.99e-02 |
| Pancreas | -0.0854 | 0.0467 | 6.74e-02 |
| Prostate | -0.1206 | 0.0573 | 3.53e-02 |
| Skin Not Sun Exposed Suprapubic | 0.2315 | 0.1012 | 2.21e-02 |
| Skin Sun Exposed Lower leg | 0.1934 | 0.0845 | 2.21e-02 |
| Small Intestine Terminal Ileum | -0.1725 | 0.1531 | 2.60e-01 |
| Stomach | 0.1453 | 0.1155 | 2.08e-01 |
| Testis | 0.1256 | 0.0482 | 9.10e-03 |
| Thyroid | 0.0406 | 0.0713 | 5.69e-01 |
| ^1^Bold values represent Bonferroni significant p-values (p ≤ 0.0017) | | | |
