# Supplementary material for: SOX7: Autism associated gene identified by analysis of multi-Omics data
Source: PLoS One. 2025 May 15;20(5):e0320096. doi: 10.1371/journal.pone.0320096 (PMC12080844; doi:10.1371/journal.pone.0320096)
Supplement: S3 Table — (DOCX) [file pone.0320096.s004.docx]

| **Supplementary Table 3.** 2-SMR results between ASD (Outcome) and *SOX7* expression (Exposure) (EUR GTEx samples only) | | | |
| --- | --- | --- | --- |
| **SOX7 Expressing Tissue** | **Beta** | **SE** | **P-value^1^** |
| Adipose Visceral Omentum | 0.2158 | 0.0994 | 2.99e-02 |
| Adrenal Gland | -0.1299 | 0.0525 | 1.34e-02 |
| Artery Aorta | 0.0807 | 0.0945 | 3.93e-01 |
| Artery Tibial | 0.1992 | 0.1155 | 8.46e-02 |
| Brain Amygdala | -0.1670 | 0.0423 | **7.74e-05** |
| Brain Anterior cingulate cortex BA24 | -0.0463 | 0.0576 | 4.21e-01 |
| Brain Caudate basal ganglia | -0.0104 | 0.0729 | 8.86e-01 |
| Brain Cerebellar Hemisphere | 0.0989 | 0.0285 | **5.31e-04** |
| Brain Frontal Cortex BA9 | 0.0102 | 0.0399 | 7.98e-01 |
| Brain Putamen basal ganglia | 0.0057 | 0.0545 | 9.16e-01 |
| Brain Spinal cord cervical c-1 | -0.1655 | 0.0645 | 1.03e-02 |
| Brain Substantia nigra | -0.0904 | 0.0318 | 4.46e-03 |
| Colon Sigmoid | 0.0458 | 0.0946 | 6.29e-01 |
| Colon Transverse | -0.2608 | 0.0981 | 7.83e-03 |
| Esophagus Gastroesophageal Junction | 0.2045 | 0.0784 | 9.10e-03 |
| Esophagus Muscularis | 0.0230 | 0.0889 | 7.96e-01 |
| Heart Atrial Appendage | -0.1378 | 0.0814 | 9.07e-02 |
| Liver | -0.0677 | 0.0316 | 3.21e-02 |
| Muscle Skeletal | -0.0887 | 0.1119 | 4.28e-01 |
| Nerve Tibial | 0.1512 | 0.0780 | 5.26e-02 |
| Pancreas | 0.0732 | 0.0394 | 6.34e-02 |
| Skin Not Sun Exposed Suprapubic | 0.2111 | 0.0977 | 3.07e-02 |
| Skin Sun Exposed Lower leg | 0.0503 | 0.0936 | 5.91e-01 |
| Small Intestine Terminal Ileum | -0.0639 | 0.0836 | 4.45e-01 |
| Spleen | 0.0120 | 0.0552 | 8.28e-01 |
| Stomach | -0.0746 | 0.1116 | 5.04e-01 |
| Testis | 0.1154 | 0.0450 | 1.04e-02 |
| Thyroid | 0.0122 | 0.0660 | 8.53e-01 |
| ^1^Bold values represent Bonferroni significant p-values (p ≤ 0.0018) | | | |
