# Supplementary material for: SOX7: Autism associated gene identified by analysis of multi-Omics data
Source: PLoS One. 2025 May 15;20(5):e0320096. doi: 10.1371/journal.pone.0320096 (PMC12080844; doi:10.1371/journal.pone.0320096)
Supplement: Attachment — Submitted filename: Point-by-point-response.docx [file pone.0320096.s006.docx]

**Reviewer #1**

In the study of Gonzales et al., the authors analyzed the GWAS and transcriptome data and identified SOX7 as an ASD risk gene. However, the results could not robustly support SOX7 as a novel risk gene for ASD. My several comments as listed below:

**We thank the reviewer for the positive comments.**

1. It is cautious to state that SOX7 is a novel ASD risk gene. Is this study the first one to support the connection between SOX7 and ASD?

**Response:** Thank the reviewer for the comments. We researched literature and did not find any publications that reported a connection between SOX7 and autism spectrum disorder (ASD). However, we removed the word “novel” to account for any publications we may have overlooked.

2. The authors did not perform any experiment to support their conclusion, how to support SOX7 as an ASD risk gene?

**Response:** We thank the reviewer for their suggestion on additional experiments to support SOX7 as a risk gene for ASD. While in vitro functional analyses were not feasible in the given time frame, we opted to perform a two-sample Mendelian Randomization using eQTLs from multiple substructures in the brain as well as whole blood to assess the causal effects of SOX7 expression on ASD diagnosis. The Causal Inference with Two-Sample Mendelian Randomization demonstrates a causal relationship between SOX7 expression and ASD. We have included the methods and results of this analysis in the Methods and Results sections of our manuscript.

The main contents of the Causal Inference with Two-Sample Mendelian Randomization are as follows:

To assess a potential causal effect of SOX7 expression on ASD, we use the R package ‘TwoSampleMR’ to conduct two-sample mendelian randomization using SOX7 eQTLs obtained from GTEx as instruments (ref 44, 45 in the manuscript). Top expression quantitative trait loci (eQTLs) for multiple tissues were obtained from the Gene-Tissue Expression (GTEx) portal, using the v8 release (refence 37 in the manuscript). The top eQTL per tissue was matched to its corresponding SNP in the ASD2019 GWAS data, palindromic SNPs that could not be inferred via minor allele frequency (MAF) as well as strand ambiguous SNPs were removed from the analysis. Additionally, effect direction was harmonized with the GWAS outcome data to ensure the same allele is referenced in both data sets. We performed single-instrument analysis, using the Wald ratio method to estimate effects of SOX7 expression in various tissues on ASD outcome. Thirty tissues were tested individually. A Bonferroni corrected threshold of $p\leq.0017$ was used to claim significance. Additionally, while the full GTEx data is majority white donors (84.6%), the v8 release includes a separate, European only subset which contains 29 tissues with a significant SOX7 eQTL. We performed a secondary analysis using this dataset, since our GWAS dataset is European only. Out of 30 tissues tested, SOX7 expression in 6 tissues were statistically significant after correcting for multiple testing in the full dataset, including 3 subregions in the brain: cerebellar hemisphere ($\beta=0.1054, p=5.31\times{10}^{-4}$), hypothalamus ($\beta= 0.1799, p=4.71\times{10}^{-4}$), and spinal cord ($\beta= 0.1997, p=8.84\times{10}^{-4}$) (Table 3 in the manuscript). Interestingly, in the European only subset, the cerebellar hemisphere remained significant ($\beta= 0.0989, p=5.31\times{10}^{-4}$) (Table 4 in the manuscript). Full results for both analyses can be found in Supplementary Tables 2 and 3 in the manuscript, respectively. These results demonstrate a causal relationship between SOX7 expression and ASD. (More details can be found in the manuscript).

3. Correlation between SOX7 expression and ASD phenotypes may be needed to support the conclusion.

**Response:** Thank the reviewer for the comments. We have added Causal Inference with Two-Sample Mendelian Randomization (MR) in this revision, which demonstrates a causal relationship between SOX7 expression and ASD. The MR analysis is designed to address causal inference rather than mere correlation. The MR uses genetic variants (eQTLs in this case) as instruments to estimate the causal effect of SOX7 expression on ASD risk. This approach helps mitigate confounding factors and reverse causation issues that could affect observational correlation analyses. The results of causal inference show that SOX7 expression levels significantly influence ASD risk in several tissues. Correlation between SOX7 expression and ASD might be implied by the results, but the MR approach provides a more robust causal inference.

4. eQTL analysis is required for ASD-associated genetic variants and SOX7 expression.

**Response:** We appreciate the reviewer’s comment regarding the need for eQTL analysis for ASD-associated genetic variants in relation to SOX7 expression. We have indeed conducted a comprehensive eQTL analysis as part of our Mendelian randomization (MR) study to investigate the causal effect of SOX7 expression on autism spectrum disorder (ASD).

*eQTL Data Integration:* We utilized the R package ‘TwoSampleMR’ to perform two-sample Mendelian randomization using eQTLs for SOX7 obtained from the Gene-Tissue Expression (GTEx) portal (v8 release). The eQTLs for SOX7 across multiple tissues were matched to corresponding SNPs in the ASD2019 GWAS data. This approach ensures that we are using relevant genetic instruments to explore the causal relationship between SOX7 expression and ASD.

*Quality Control and Harmonization:* To ensure robustness in our analysis, we removed palindromic SNPs and strand-ambiguous SNPs that could not be inferred using minor allele frequency (MAF). Additionally, we harmonized effect directions between the eQTL data and the GWAS outcome data, aligning alleles to maintain consistency across datasets.

*Analysis and Significance:* We conducted single-instrument analyses for SOX7 expression in 30 tissues, applying the Wald ratio method to estimate the effects on ASD. After correcting for multiple testing using a Bonferroni threshold (p ≤ 0.0017), we found significant associations in six tissues, including three brain subregions (cerebellar hemisphere, hypothalamus, and spinal cord). Notably, SOX7 expression in the cerebellar hemisphere remained significant in a secondary analysis using a European-only subset of the GTEx data, aligning with the European-only ASD GWAS dataset.

*Conclusion:* These findings, detailed in Table 3 and Table 4 of our manuscript, along with Supplementary Tables 2 and 3, support a causal relationship between SOX7 expression and ASD. The analysis of eQTLs for ASD-associated variants and SOX7 expression was crucial in demonstrating this causal link.

**Reviewer #2**

The authors performed gene-based association test and DEG test flawlessly. However, there is one principal issue regarding to the GWAS set used and subsequent conclusions made: the PGC ASD 2017 and PGC ASD 2019 dataset are cumulative (i.e., the raw data used in ASD 2019 is a superset of ASD 2017, and I can confirm this with confidence as a PGC panel member). Therefore, the conclusion that SOX7 association has been found in both ASD 2017 and 2019 dataset is unsurprising and statistically expected. More importantly, these two datasets cannot be claimed as replicates for their inclusion (Page 14, results).

**We thank the reviewer for the positive comments.**

1. It is recommended that a "true" replicate of gene-based association test to be performed using an independent ASD dataset such as SPARK (Matoba et al. 2020 https://www.nature.com/articles/s41398- 020-00953-9). I believe it should not take too much effort to repeat the analysis and the conclusions would be significantly consolidated.

**Response:** We appreciate the reviewer’s suggestion to validate our findings using an independent ASD dataset, such as SPARK. We acknowledge the importance of replication in strengthening our conclusions.

In response to the recommendation, we performed additional gene based GWAS analyses using the SPARK dataset (Matoba et al., 2020). However, due to the heterogeneity and the nonsignificant SNPs located near SOX7 in SPARK, we were unable to replicate the results in this particular dataset. Table 1 below shows the p value, test statistic, effect size, and other information of each SNP on gene SOX7 in SPARK, 2017asd and 2019asd datasets. All the p value of SNPs in SPARK data are very large. Therefore, it is possible that gene based study can indicate any significance in SOX7.

**Table 1. Genetic effects and significance of SNPs in gene SOX7 among three datasets**

| **SNP** | **CHR** | **BP** | $\boldsymbol{\beta}$ | **SE** | **Z** | **P** |
| --- | --- | --- | --- | --- | --- | --- |
| ASD 2017 | | | | | | |
| rs10100209 | 8 | 10584212 | -0.088 | 0.028 | -3.055 | 0.002 |
| rs11250064 | 8 | 10581882 | -0.076 | 0.029 | -2.545 | 0.01 |
| rs4841432 | 8 | 10583506 | 0.107 | 0.029 | 3.704 | 0.0002 |
| rs7005905 | 8 | 10585144 | -0.096 | 0.028 | -3.364 | 0.0007 |
| rs7009920 | 8 | 10585162 | 0.068 | 0.033 | 2.059 | 0.03 |
| rs7836366 | 8 | 10585683 | -0.09 | 0.029 | -3.168 | 0.001 |
| ASD 2019 | | | | | | |
| rs10100209 | 8 | 10584212 | -0.054 | 0.014 | -3.859 | 0.0001 |
| rs11250064 | 8 | 10581882 | -0.052 | 0.015 | -3.500 | 0.0004 |
| rs4841432 | 8 | 10583506 | 0.081 | 0.014 | 5.456 | 4.40E-08 |
| rs7005905 | 8 | 10585144 | -0.056 | 0.014 | -4.006 | 6.44E-05 |
| rs7009920 | 8 | 10585162 | 0.072 | 0.016 | 4.400 | 1.11E-05 |
| rs7836366 | 8 | 10585683 | -0.055 | 0.014 | -3.88 | 9.84E-05 |
| SPARK | | | | | | |
| rs10100209 | 8 | 10584212 | -0.017 | 0.031 | -0.566 | 0.571 |
| rs11250064 | 8 | 10581882 | -0.004 | 0.032 | -0.154 | 0.877 |
| rs4841432 | 8 | 10583506 | 0.043 | 0.032 | 1.380 | 0.167 |
| rs7005905 | 8 | 10585144 | -0.016 | 0.031 | -0.543 | 0.586 |
| rs7009920 | 8 | 10585162 | -0.002 | 0.035 | -0.081 | 0.935 |
| rs7836366 | 8 | 10585683 | -0.014 | 0.030 | -0.469 | 0.638 |

We also investigate the overlap of individuals in the ASD 2019 and ASD 2017. From the Table 2, we can see that 71% of the ASD 2019 data does not have an overlap with ASD 2017 data. We can consider that 2017 ASD partially replicated gene SOX7 which was identified from 2019 ASD data.

**Table 2. Components and overlap between ASD 2017 and ASD 2019 datasets**

| **Consortium** | **Design** | **ASD 2017** | **ASD 2019** | **ASD 2017** | **ASD 2019** |
| --- | --- | --- | --- | --- | --- |
| iPSYCH | Case-Control | 0 | 35740 | NO | YES |
| Geschwind Autism Center of Excellence (ACE) | Trio | 744 | 782 | YES | YES |
| Autism Genome Project (AGP) | Trio | 4400 | 4544 | YES | YES |
| Autism Genetic Resource Exchange (AGRE) | Trio | 3234 | 1948 | YES | YES |
| NIMH Repository and Montreal/Boston Collection (MonBos) | Trio | 234 | 2792 | YES | YES |
| Simons Simplex Collection (SSC) | Trio | 4378 | 4462 | YES | YES |
| Finnish Case-Control ASD Collection | Case-Control | 685 | 0 | YES | NO |
| Weiss Laboratory Autism Collection | Case-Control | 774 | 0 | YES | NO |
| Population-Based Autism Genetics and Environment Study (PAGES) | Case-Control | 1505 | 0 | YES | NO |
| Total |  | 15954 | 50268 |  | 71.1%=35740/50268 |

Despite this limitation, we have rigorously analyzed the relationship between SOX7 expression and ASD using the following approach:

*Causal Inference with Two-Sample Mendelian Randomization:* We utilized the R package ‘TwoSampleMR’ to conduct two-sample Mendelian randomization with SOX7 eQTLs obtained from the GTEx portal (v8 release). This approach allowed us to leverage the genetic instruments for SOX7 expression to estimate its causal effect on ASD.

*eQTL Data and Analysis:* Top eQTLs for SOX7 across multiple tissues were matched to SNPs in the ASD2019 GWAS data. We ensured the robustness of our analysis by removing palindromic SNPs and strand-ambiguous SNPs, and harmonizing effect directions between the eQTL and GWAS datasets. Single-instrument analyses using the Wald ratio method were conducted across 30 tissues, with a Bonferroni-corrected significance threshold of p ≤ 0.0017.

*Significant Findings:* Our analysis identified significant associations between SOX7 expression and ASD in six tissues, including three brain subregions (cerebellar hemisphere, hypothalamus, and spinal cord). Notably, the cerebellar hemisphere remained significant in a secondary analysis using the European-only subset of GTEx data, aligning with the European-only ASD GWAS dataset. Detailed results can be found in Table 3 and Table 4 of the manuscript, as well as Supplementary Tables 2 and 3.

*Conclusion:* Although the SPARK dataset did not provide replicable results due to some limitations, our comprehensive analysis using the GTEx v8 release and the ASD2019 GWAS data robustly supports a causal relationship between SOX7 expression and ASD. We believe this multi-faceted approach provides strong evidence for our conclusions.

We hope this addresses the reviewer’s concerns and further consolidates the validity of our findings.

**Reviewer #3**

In the study conducted by Samantha Gonzales and colleagues, utilizing gene-based analysis and two extensive GWAS datasets from 2019 and 2017, identifying five genes with a substantial link to autism spectrum disorder (ASD). Further, integrated two ASD and control postmortem brain gene expression data revealed that the SOX7, an (SRY-related HMG-box) family of transcription factors as a high-confidence risk gene upregulated in ASD patients. The design of this study is straightforward, but the description of the results needs to be significantly improved. For example, the scientific rationale of each section within the results needs to be more thoroughly explained. I have the following comments:

**We thank the reviewer for the positive comments.**

1. A major concern of this study is the reliance on two large GWAS datasets from the Psychiatric Genomics Consortium (PGC). The dataset used for discovery, dated 2019, is not independent of the dataset used for replication, dated 2017.

**Response:** We thank the reviewer for the comments. We investigated the overlap of individuals in the ASD 2019 and ASD 2017. From the Table 2, we can see that 71% of the ASD 2019 data does not have an overlap with ASD 2017 data. We can consider that 2017 ASD partially replicated gene SOX7 which was identified from 2019 ASD data.

Table 2. Components and overlap between ASD 2017 and ASD 2019 datasets

| **Consortium** | **Design** | **ASD 2017** | **ASD 2019** | **ASD 2017** | **ASD 2019** |
| --- | --- | --- | --- | --- | --- |
| iPSYCH | Case-Control | 0 | 35740 | NO | YES |
| Geschwind Autism Center of Excellence (ACE) | Trio | 744 | 782 | YES | YES |
| Autism Genome Project (AGP) | Trio | 4400 | 4544 | YES | YES |
| Autism Genetic Resource Exchange (AGRE) | Trio | 3234 | 1948 | YES | YES |
| NIMH Repository and Montreal/Boston Collection (MonBos) | Trio | 234 | 2792 | YES | YES |
| Simons Simplex Collection (SSC) | Trio | 4378 | 4462 | YES | YES |
| Finnish Case-Control ASD Collection | Case-Control | 685 | 0 | YES | NO |
| Weiss Laboratory Autism Collection | Case-Control | 774 | 0 | YES | NO |
| Population-Based Autism Genetics and Environment Study (PAGES) | Case-Control | 1505 | 0 | YES | NO |
| Total |  | 15954 | 50268 |  | 71.1%=35740/50268 |

Despite this limitation, we have rigorously analyzed the relationship between SOX7 expression and ASD using the following approach:

*Causal Inference with Two-Sample Mendelian Randomization:* We utilized the R package ‘TwoSampleMR’ to conduct two-sample Mendelian randomization with SOX7 eQTLs obtained from the GTEx portal (v8 release). This approach allowed us to leverage the genetic instruments for SOX7 expression to estimate its causal effect on ASD.

*eQTL Data and Analysis:* Top eQTLs for SOX7 across multiple tissues were matched to SNPs in the ASD2019 GWAS data. We ensured the robustness of our analysis by removing palindromic SNPs and strand-ambiguous SNPs, and harmonizing effect directions between the eQTL and GWAS datasets. Single-instrument analyses using the Wald ratio method were conducted across 30 tissues, with a Bonferroni-corrected significance threshold of p ≤ 0.0017.

*Significant Findings:* Our analysis identified significant associations between SOX7 expression and ASD in six tissues, including three brain subregions (cerebellar hemisphere, hypothalamus, and spinal cord). Notably, the cerebellar hemisphere remained significant in a secondary analysis using the European-only subset of GTEx data, aligning with the European-only ASD GWAS dataset. Detailed results can be found in Table 3 and Table 4 of the manuscript, as well as Supplementary Tables 2 and 3.

*Conclusion:* Although the SPARK dataset did not provide replicable results due to some limitations, our comprehensive analysis using the GTEx v8 release and the ASD2019 GWAS data robustly supports a causal relationship between SOX7 expression and ASD. We believe this multi-faceted approach provides strong evidence for our conclusions.

We hope this addresses the reviewer’s concerns and further consolidates the validity of our findings.

2. Please combine Figures 2a and 2b as one figure. Additionally, please describe the various panels within this figure, clarifying terms such as “all sample” and “white sample.” 3. Using gene expression counts in Figures 2a and 2b creates confusion, particularly as Figure 2b is presented on different scales, please address this for clarity.

**Response:** We appreciate the reviewer's suggestion to combine Figures 2a and 2b for clarity. We have merged these figures into a single figure, now referred to as Figure 2, with clearly labeled panels.
