# Supplementary material for: SOX7: Autism associated gene identified by analysis of multi-Omics data
Source: PLoS One. 2025 May 15;20(5):e0320096. doi: 10.1371/journal.pone.0320096 (PMC12080844; doi:10.1371/journal.pone.0320096)
Supplement: Attachment — Submitted filename: Point-by-point-response_auresp_2.docx [file pone.0320096.s007.docx]

Reviewers' comments:

Reviewer's Responses to Questions

**Comments to the Author**

1. If the authors have adequately addressed your comments raised in a previous round of review and you feel that this manuscript is now acceptable for publication, you may indicate that here to bypass the “Comments to the Author” section, enter your conflict of interest statement in the “Confidential to Editor” section, and submit your "Accept" recommendation.

Reviewer #1: All comments have been addressed

Reviewer #2: All comments have been addressed

Reviewer #3: All comments have been addressed

2. Is the manuscript technically sound, and do the data support the conclusions?

The manuscript must describe a technically sound piece of scientific research with data that supports the conclusions. Experiments must have been conducted rigorously, with appropriate controls, replication, and sample sizes. The conclusions must be drawn appropriately based on the data presented.

Reviewer #1: Yes

Reviewer #2: Yes

Reviewer #3: Yes

3. Has the statistical analysis been performed appropriately and rigorously?

Reviewer #1: Yes

Reviewer #2: (No Response)

Reviewer #3: Yes

4. Have the authors made all data underlying the findings in their manuscript fully available?

The [PLOS Data policy](https://urldefense.com/v3/__http:/c05y1x9s.r.us-east-2.awstrack.me/L0/http:*2F*2Fwww.plosone.org*2Fstatic*2Fpolicies.action*23sharing/1/010f019264838652-743ca6bb-2171-43dd-a1d1-2042655e0091-000000/VnS0ZWXZZ4nwPwTvtzlfK7L4zBQ=179__;JSUlJSU!!FjuHKAHQs5udqho!N-WLKUjxkwBsKOQKCvt8cMZNxgRulh0_QzDkTkBS9TpIgkK_sVqaF1tqvhLrqIjdP5blX5r5SmDDISLmb1IdhPrC$) requires authors to make all data underlying the findings described in their manuscript fully available without restriction, with rare exception (please refer to the Data Availability Statement in the manuscript PDF file). The data should be provided as part of the manuscript or its supporting information, or deposited to a public repository. For example, in addition to summary statistics, the data points behind means, medians and variance measures should be available. If there are restrictions on publicly sharing data—e.g. participant privacy or use of data from a third party—those must be specified.

Reviewer #1: Yes

Reviewer #2: Yes

Reviewer #3: Yes

5. Is the manuscript presented in an intelligible fashion and written in standard English?

PLOS ONE does not copyedit accepted manuscripts, so the language in submitted articles must be clear, correct, and unambiguous. Any typographical or grammatical errors should be corrected at revision, so please note any specific errors here.

Reviewer #1: Yes

Reviewer #2: Yes

Reviewer #3: Yes

6. Review Comments to the Author

Please use the space provided to explain your answers to the questions above. You may also include additional comments for the author, including concerns about dual publication, research ethics, or publication ethics. (Please upload your review as an attachment if it exceeds 20,000 characters)

Reviewer #1: The authors have addressed my concerns. However, the abstract contains too much description about the background knowledge. Major results should be briefly summarized rather than listed all the results in the abstract.
The abstract is suggested to be revised before publication. I have no further comments.

Reviewer #2: There is one minor issue that needs to be amended before allowing publication: in Fig. 2, please plot all data points in addition to the box-whisker plot.

**Response:** We thank the reviewer for the insightful suggestions. We have updated Figure 2 as suggested in the updated manuscript.

Reviewer #3: The authors have done an excellent job responding to my criticisms. I am happy to now recommend this manuscript for publication in Plos One.

7. PLOS authors have the option to publish the peer review history of their article ([what does this mean?](https://urldefense.com/v3/__https:/c05y1x9s.r.us-east-2.awstrack.me/L0/https:*2F*2Fjournals.plos.org*2Fplosone*2Fs*2Feditorial-and-peer-review-process*23loc-peer-review-history/1/010f019264838652-743ca6bb-2171-43dd-a1d1-2042655e0091-000000/GHNpyab6_F74YTwuzT6NsDtm1yo=179__;JSUlJSUl!!FjuHKAHQs5udqho!N-WLKUjxkwBsKOQKCvt8cMZNxgRulh0_QzDkTkBS9TpIgkK_sVqaF1tqvhLrqIjdP5blX5r5SmDDISLmb49mlUAX$)). If published, this will include your full peer review and any attached files.


If you choose “no”, your identity will remain anonymous but your review may still be made public.


**Do you want your identity to be public for this peer review?** For information about this choice, including consent withdrawal, please see our [Privacy Policy](https://urldefense.com/v3/__https:/c05y1x9s.r.us-east-2.awstrack.me/L0/https:*2F*2Fwww.plos.org*2Fprivacy-policy/1/010f019264838652-743ca6bb-2171-43dd-a1d1-2042655e0091-000000/rTJ1ek9UlFHXMhgAyQcyp8IdNFg=179__;JSUl!!FjuHKAHQs5udqho!N-WLKUjxkwBsKOQKCvt8cMZNxgRulh0_QzDkTkBS9TpIgkK_sVqaF1tqvhLrqIjdP5blX5r5SmDDISLmb1Na0SuC$).

Reviewer #1: No

Reviewer #2: **Yes:** Siwei Zhang

Reviewer #3: No
